# Comparative analysis of gut microbiota in elderly people of urbanized towns and longevity villages

**DOI:** 10.1186/s12866-015-0386-8

**Published:** 2015-02-26

**Authors:** Se-Hoon Park, Kyung-Ah Kim, Young-Tae Ahn, Jin-Ju Jeong, Chul-Sung Huh, Dong-Hyun Kim

**Affiliations:** R &B D Center, Korea Yakult Co., Ltd., Yongin-si, Kyunggi-do 446-901 Korea; Department of Life and Nanopharmaceutical Sciences, College of Pharmacy, 1, Hoegi, Dongdaemun-ku, Seoul, 130-701 Korea; Agricultural Biotechnology, College of Agriculture and Life Sciences, Seoul National University, Seoul, 151-742 Korea

**Keywords:** Longevity, Ageing, Intestinal microbiota, LPS, *Faecalibacterium spp*

## Abstract

**Background:**

To understand differences in the gut microbiota between elderly people of urbanized town communities (UTC) and longevity village communities (LVC), we analyzed fecal microbiota collected from individuals living in 2 UTC (Seoul and Chuncheon) and 3 LVC (Gurye, Damyang, and Soonchang) selected on the basis of indices for superlongevity (the ratio of centenarians to the total population) and longevity (the ratio of those aged 85 years or greater to those aged 65 years or greater) in South Korea by 454 pyrosequencing.

**Results:**

Taxonomy-based analysis showed that The relative abundance of Firmicutes, Tenericutes, and Actinobacteria was significantly lower in LVC than in UTC. Due to an increase of Firmicutes and a reduction of Bacteroidetes, the ratio of Firmicutes to Bacteroidetes in the gut microbiota was greater in UTC adults than in UTC children or LVC adults. The population levels of *Bacteroides, Prevotella*, and *Lachnospira* were significantly higher in LVC than in UTC, but the levels of *Dialister, Subdoligranulum, Megamonas,* EF401882_g*,* and AM275436_g were lower in LVC than in UTC. Although most of the species detected in LVC were detected in UTC, some *Bacteroides* spp. and *Faecalibacterium* spp. were detected only in LVC. Among *Bacteroides* spp., ACWH_s, EF403317_s, and EF403722_s were detected in children and LVC samples only but FJ363527_s, 4P000677_s, and 4P000015_s were detected in UTC samples. EF402172_s and EF404388_s, members of *Faecalibacterium* spp., which are known to have anti-inflammatory properties, were detected in LVC and children only (>3.9% of total sequence). In addition, the fecal lipopolysaccharides (LPS) content was significantly higher in UTC than in LVC.

**Conclusions:**

These findings suggest that maintaining gut microbiota, including *Faecalibacterium* spp. EF402172_s and EF404388_s, as well as low LPS levels may play an important role in preserving residents’ health in LVC.

**Electronic supplementary material:**

The online version of this article (doi:10.1186/s12866-015-0386-8) contains supplementary material, which is available to authorized users.

## Background

In the last 50 years, the mean human life expectancy in most developed countries, including South Korea, has increased from approximately 50 to 80 years, with women having a higher mean life expectancy than do men [1]. While genetic factors are the most important determinants of lifespan, it has been suggested that environmental factors such as diet also influence lifespan [2].

During gestation, the fetus develops and grows in the sterile environment of the mother’s uterus. During delivery and immediately afterward, the infant’s external surfaces, such as the gut and skin, are colonized by microbes transmitted from the mother, the environment, and the diet. Such colonization is pivotal to development of the gastrointestinal mucosa and the immune system [3,4]. In contrast to the typical viewpoint that healthy intestinal microbiota are relatively stable throughout adulthood, intestinal microbiota are disturbed by exogenous and endogenous factors such as diet, antibiotics, and stress [5,6]. For example, high-fat diets lead to increased levels of gram-negative bacteria that induce lipopolysaccharide production in the intestine, which can lead to inflammation, obesity, and cancer [7-9]. While development of beneficial and stable intestinal microbiota during infancy and childhood is of great interest, a few studies have focused on genetic and physiological factors that affect intestinal microbiota during ageing and on the impact of these modifications on health and longevity [10-12]. Recent reports employed advanced molecular characterization techniques such as 16S rRNA amplicon sequencing to study intestinal microbiota in ageing individuals, focusing on variability of intestinal microbiota among older subjects and on different effects of ageing, lifestyle, and dietary habits on intestinal microbiota [13,14].

Previous studies have not considered potential differences in gut microbiota between elderly people from urbanized town communities (UTC) and those from longevity village communities (LVC). In this study, we analyzed fecal microbiota collected from individuals living in 2 UTC (Seoul and Chuncheon) and 3 LVC (Gurye, Damyang, and Sunchang), selected on the basis of indices for superlongevity (the ratio of centenarians to the total population) and longevity (the ratio of those aged 85 years or greater to those aged 65 years or greater), as reported by the Korea National Statistical Office [15].

## Methods

### Subjects

Nine of 170 counties in South Korea, including 7 longevity (Centenarian) rural areas and 2 urbanized downtown areas, were investigated according to the methods described by Park et al. [16]. The LVC selected were Damyang (Jeonranam-do), Gurye (Jeonranam-do), and Sunchang (Jeonrabug-do). The mean age of the LVC individuals was 69.96 ± 11.14 y and included 27 men and 42 women (Table 1). The male age group distribution was: 2 (40–49 y), 4 (50–50 y), 11 (60–69 y), 10 (over 70 y). The female age group distribution was: 4 (50–59 y), 9 (60–69 y), 29 (over 70 y). They were selected on the basis of indices for superlongevity (the ratio of centenarians to the total population) and longevity (the ratio of those aged 85 years or greater to those aged 65 years or greater), as reported by the Korea National Statistical Office [15]. LVC were chosen with a superlongevity ratio greater than 20 centenarians per 100,000 populations and a longevity ratio greater than 7%. Urbanized South Korean communities chosen for the study were Seoul, with a population greater than 9.50 million, and Chuncheon with populations greater than 0.25 million. The mean age of the UTC adults was 53.00 ± 8.47 y and included 19 men and 21 women. The male age group distribution was: 8 (40–49 y), 7 (50–59 y), and 4 (60–69 y). The female age group distribution was: 8 (40–49 y), 6 (50–59 y), and 7 (60–69 y). The mean age of UTC children was 8.5 ± 2.8 y and consisted of 18 boys (age 0–8 y) and 4 girls (age 0–8 y). Individuals who were under medication, especially those that took antibiotics regularly or during the time of the study, were excluded. Recruitment and collection of stool samples were approved by the Committee for the Care and Use of Clinical Study in the Medical School, Kyung Hee University (KMC IRB 0922-08-A1). Enrolled subjects were recruited from Kyung Hee University Medical Center, Seoul, Republic of Korea and provided written informed consent to participate in the study. As shown in Table 1, no significant difference between UTC and LVC in the characteristics of the study subjects was found.

**Table 1:** **Characteristics of the study subjects**

		**UTC**				**LVC**			
		**Children**	**40′s**	**50′s**	**60′s**	**40′s**	**50′s**	**60′s**	**>70**
**Age**		8.5 ± 2.8	44.9 ± 2.5	54.6 ± 2.0	64.5 ± 2.8	44.5 ± 2.1	54.5 ± 1.6	66.0 ± 2.5	80.0 ± 9.0
**n**		22	16	13	11	2	8	20	39
**Sex(male/female)**		18/4	8/8	7/6	4/7	2/0	4/4	11/9	10/29
**Height (m)**		1.34 ± 0.18	1.62 ± 0.07	1.65 ± 0.09	1.57 ± 0.06	1.75 ± 0.07	1.61 ± 0.08	1.60 ± 0.07	1.52 ± 0.09
**Weight (kg)**		33.9 ± 10.8	61.8 ± 11.2	61.1 ± 7.8	55.6 ± 6.1	76.5 ± 2.12	56.8 ± 4.1	60.7 ± 8.7	48.7 ± 8.1
**BMI (kg/m** ^**2**^ **)**		18.1 ± 2.6	23.4 ± 3.2	22.5 ± 2.3	23.7 ± 1.7	25.0 ± 1.3	21.9 ± 1.5	23.6 ± 2.7	21.0 ± 2.5
	>25	0	5	5	2	1	0	6	3
	<25	22	11	8	9	1	8	14	36
**Diet**									
	Regular	22	14	11	10	2	8	20	17
	Vegetarian	0	2	2	1	0	0	0	0
**Alcohol consumption**									
	Yes	0	10	10	3	1	4	7	7
	No	22	6	3	8	1	4	13	11
**Smoking**									
	Yes	0	5	4	2	0	2	1	0
	No	22	11	9	9	2	6	19	39

Values are expressed as mean±SD.

### DNA extraction, pyrosequencing, and data analysis

Genomic DNA was extracted from fecal samples using a commercial DNA isolation kit (QIAamp DNA Stool Mini Kit, Qiagen, Hilden, Germany) following the manufacturer’s protocol. Amplification of genomic DNA was performed using barcoded primers that targeted the V1 to V3 regions of the bacterial 16S rRNA gene. The amplification, sequencing, and basic analysis were performed according to methods described by Chun *et al.* [17] and completed by ChunLab Inc. (Seoul, Korea) by using a 454 GS FLX Titanium Sequencing System (Roche, Branford, CT, USA). Sequences for each sample were sorted by a unique barcode and low quality reads (average quality score <25 or read length <300 bp) were removed. Sequence reads were identified using the EzTaxon-e database (http://eztaxon-e.ezbiocloud.net/) on the basis of 16S rRNA sequence data [9,18]. The number of sequences analyzed, observed diversity richness [Operational Taxonomic Units (OTUs)], estimated OTU richness (ACE and Chao1), and pyrosequencing coverage indicated in Additional file 1: Table S1 were calculated using the Mothur program and defined considering a cut-off value of 97% similarity of the 16S rRNA gene sequences. 454 pyrosquencing reads have been deposited in the NCBI’s short read archive under accession number SRP052893.

### Limulus amoebocyte lysate assay

Fecal endotoxin contents were determined by using the Diazo-coupled limulus amoebocyte lysate (LAL) assays (Cape Cod Inc., E. Falmouth, MA) according to manufacturer’s protocol. Briefly, fresh stools were carefully suspended in 9-volumes of PBS in a pyrogen-free tube and sonicated for 1 hr on ice [19]. After centrifugation at 400 g for 15 min, the upper 10 ml was collected, sterilized by filtration through a 0.45 μm filter followed by re-filtration through a 0.22 μm filter, and inactivated for 10 min at 70°C. Filtered sonicate was then incubated with LAL solution to continue the analysis.

### Statistical analysis

The data are expressed as the means ± standard deviations. Statistical analysis of the data was performed with One-way analysis of variance (ANOVA). Differences with a p < 0.05 were considered to be statistically significant.

## Results

We used pyrosequencing to analyze gut microbiota compositions in fecal samples from UTC adults and children and LVC adults. The rarefaction curves (Additional file 2: Figure S1), number of sequences analyzed, and estimated operational taxonomic unit (OTU) richness (Additional file 1: Table S1) indicated that the bacterial richness and diversity between LVC and UTC was not significantly different.

**Figure 1:**
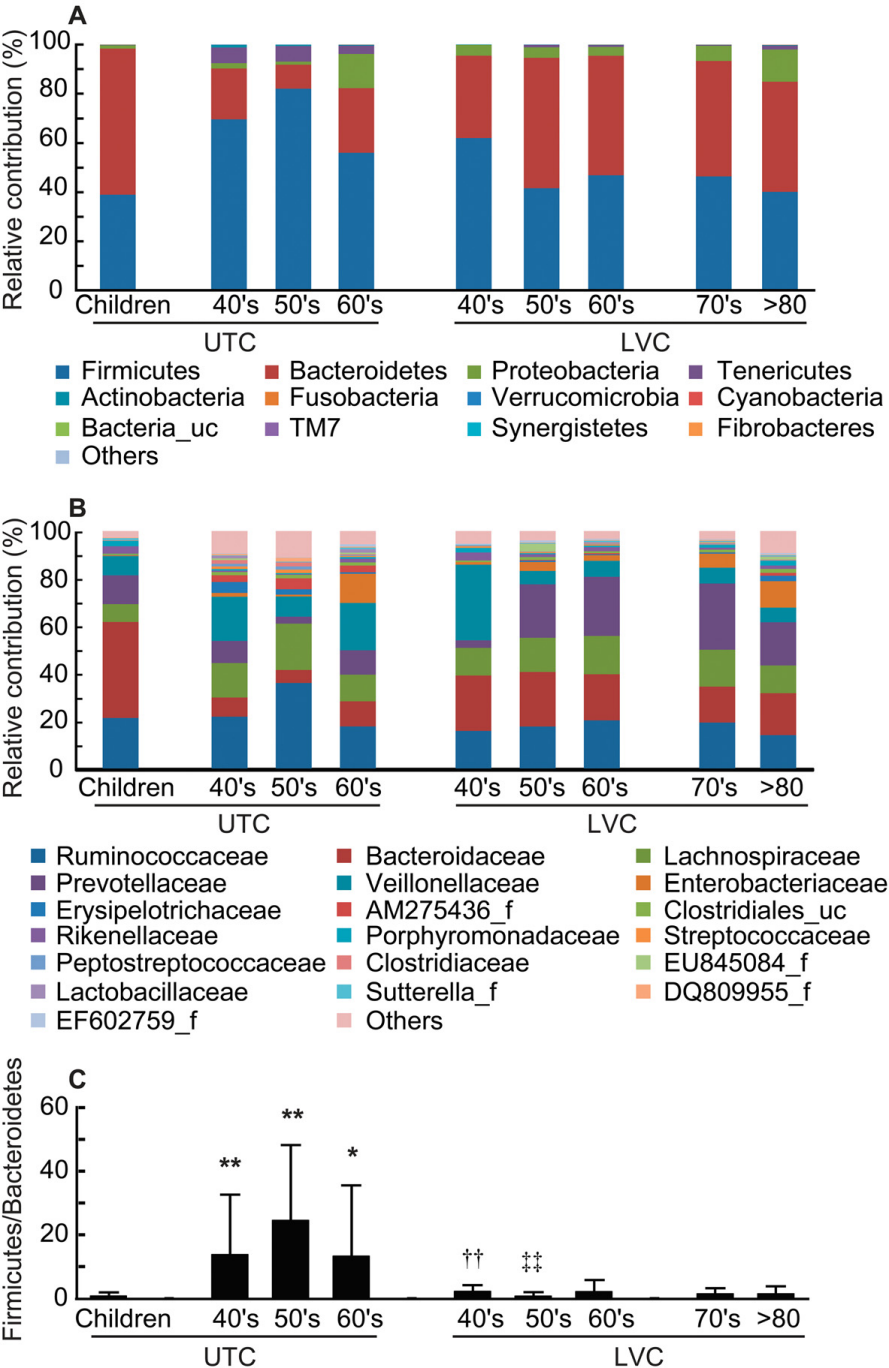
**The composition of intestinal microbiota of UTC and LVC.** The relative contributions of dominant phyla **(A)**, families **(B)**, and the *Firmicutes* to *Bacteroidetes* ratio **(C)** identified from pyrosequencing data are shown. LVC, longevity village communities; UTC, urbanized town communities. All values are indicated as the mean ± SD. *, *p* < 0.1 and **, *p <* 0.05 compared with UTC children; ††, *p* < 0.05 compared with UTC 40′s; ‡‡, *p* < 0.05 compared with UTC 50′s.

Taxonomy-based analysis showed that the distributions of the major phyla (Firmicutes, Bacteroidetes, and Proteobacteria) were consistent with the results of previous human gut studies (Figure 1A). However, the main dominant phyla identified in LVC were Firmicutes, Bacteroidetes, and Proteobacteria, whereas those in UTC were Firmicutes, Bacteroidetes, Tenericutes, and Proteobacteria. The relative abundance of Firmicutes, Tenericutes, and Actinobacteria was significantly lower in LVC than in UTC (40–69 y, p < 0.001, p < 0.05, and p < 0.05, respectively). The relative abundance of Firmicutes, Bacteroidetes, and Proteobacteria in LVC was similar to those in children. Due to an increase of Firmicutes and a reduction of Bacteroidetes, the ratio of Firmicutes to Bacteroidetes in the gut microbiota was greater in UTC adults than in UTC children or LVC adults (Figure 1C). At the family level, the three most abundant genera in both LVC and UTC were Ruminococcaceae, Bacteroidaceae, and Lachnospiraceae (Figure 1B). The relative abundance of Bacteroidaceae in LVC was higher than that in UTC (40–69 y, p < 0.005). At the genus level, the 6 most abundant genera were *Bacteroides*, *Prevotella*, *Faecalibacterium*, *Dialister*, *Roseburia*, and *Subdoligranulum*, which accounted for an average of 50% of the sequences (Table 2). The relative abundance of *Bacteroides*, *Prevotella*, and *Lachnospira* in LVC was higher than those in UTC but that of *Subdoligranulum, Ruminococcaceae_uc, Megamonas, Clostridiales_uc.g,* AM275436_g (*Mollicutes*), *Blautia, Clostridium_g4,* and *Eubacterium_g9* in LVC was lower than that in UTC (40–69 y, p < 0.05).

**Table 2:** **Difference in the composition (percent of total sequences) of fecal bacterial genera isolated from UTC and LVC samples**

**Genus**		**Composition** ^*^ **(%)**		
		**UTC**	**LVC**	
	**Children**	**40-69 y**	**40-69 y**	**> 70**
*Bacteroides*	40.61 ± 21.87^a^	7.05 ± 10.95^b^	21.89 ± 24.00^c^	15.84 ± 20.47^c^
*Prevotella*	11.44 ± 24.87 ^ac^	7.63 ± 14.46^a^	22.98 ± 29.61^bc^	23.54 ± 26.13^bc^
*Faecalibacterium*	10.87 ± 16.52^a^	8.99 ±11.13^a^	9.38 ± 10.64^a^	5.46 ± 7.83^a^
*Dialister*	5.10 ±10.31^ab^	10.66 ± 16.18^b^	5.07 ± 8.76^ab^	4.68 ± 7.75^a^
*Roseburia*	1.55 ± 2.28^a^	3.66 ± 3.10^b^	3.51 ± 3.67^b^	3.30 ± 3.81^b^
*Subdoligranulum*	0.88 ± 1.38^a^	4.31 ± 4.32^b^	0.57 ± 0.65^a^	0.61 ± 1.19^a^
*Ruminococcaceae_uc*	1.53 ± 1.82^a^	3.20 ± 3.03^b^	1.87 ± 1.67^a^	2.25 ± 1.99^ab^
*Lachnospira*	1.92 ± 2.51^a^	1.57 ± 2.96^a^	3.65 ± 3.30^bc^	3.28 ± 3.87^ac^
*Clostridium_g9*	1.21 ± 0.73^a^	1.76 ± 2.96^a^	1.53 ± 1.90^a^	1.14 ± 1.34^a^
*DQ905034_g*	1.56 ± 3.99^a^	1.21 ± 202^a^	0.89 ± 1.44^a^	1.19 ± 1.39^a^
*Megamonas*	0.75 ± 1.75^ac^	1.95 ± 5.56^ab^	0.19 ± 0.52^c^	0.04 ± 0.23^c^
*Clostridiales_uc_g*	0.57 ± 0.61^a^	1.44 ± 1.11^b^	0.91 ± 0.79^ad^	1.42 ± 1.45^bd^
*EF401882_g*	0.90 ± 2.38^a^	1.40 ± 2.81^a^	0.51 ± 1.17^a^	1.24 ± 3.85^a^
*EF404805_g*	0.69 ± 1.00^a^	1.50 ± 1.98^b^	0.94 ± 1.00^ab^	1.15 ± 1.11^ab^
*Escherichia*	0.24 ± 0.56^a^	1.27 ± 5.66^ab^	1.82 ± 4.50^ac^	4.00 ± 10.86^bc^
*EF403870_g*	0.67 ± 1.48^a^	0.85 ± 1.34^a^	1.06 ± 0.96^a^	1.66 ± 2.41^a^
*AM275436_g*	0.03 ± 0.11^a^	2.18 ± 4.12^b^	0.36 ± 1.11^ad^	0.37 ± 1.03^cd^
*Alistipes*	2.92 ± 4.10^a^	0.71 ± 1.80^bc^	1.66 ± 2.78^ac^	1.01 ± 1.40^bc^
*Ruminococcus*	2.42 ± 5.87^a^	1.33 ± 1.92^a^	1.04 ± 1.33^a^	0.69 ± 1.01^a^
*Clostridium_g12*	0.85 ± 1.19^a^	0.79 ± 0.85^a^	1.14 ± 1.47^a^	0.74 ± 0.75^a^
*Lachnospiraceae_uc*	0.37 ± 0.40a	1.16 ± 1.25b	0.79 ± 0.68b	0.95 ± 0.84b
*Streptococcus*	0.09 ± 0.20^ab^	1.10 ± 3.19^b^	0.46 ± 1.19^ab^	0.08 ± 0.16^a^
*Megasphaera*	1.06 ± 3.45^a^	0.82 ± 1.92^a^	0.50 ± 2.50^a^	0.19 ± 0.63^a^
*Veillonella*	0.35 ± 0.52^a^	0.87 ± 1.98^a^	0.55 ± 1.44^a^	0.70 ± 3.26^a^
*Catenibacterium*	0.03 ± 0.11^a^	1.65 ± 8.02^a^	0.03 ± 0.07^a^	0.87 ± 5.18^a^
*Parabacteroides*	1.77 ± 2.53^a^	0.48 ± 0.81^bc^	0.52 ± 0.58^bc^	0.93 ± 1.38^ac^
*Blautia*	0.10 ± 0.07^a^	0.86 ± 1.30^b^	0.34 ± 0.46^c^	0.28 ± 0.55^c^
*Clostridium_g4*	0.12 ± 0.13^a^	0.78 ± 1.44^b^	0.19 ± 0.32^a^	0.23 ± 0.37^a^
*Eubacterium_g9*	0.003 ± 0.02^a^	0.88 ± 1.42^b^	0.32 ± 0.75^c^	0.27 ± 0.97^a^
*Clostridium*	0.03 ± 0.05^a^	1.28 ± 3.89^bc^	0.19 ± 0.33^c^	0.28 ± 0.83^ac^

^*^, Mean ±SD, data with different superscript letters are significantly different (P<0.05) according to ANOVA one-way statistical analysis.

LVC, longevity village communities; UTC, urbanized town communities; nd, not detected.

**Figure 2:**
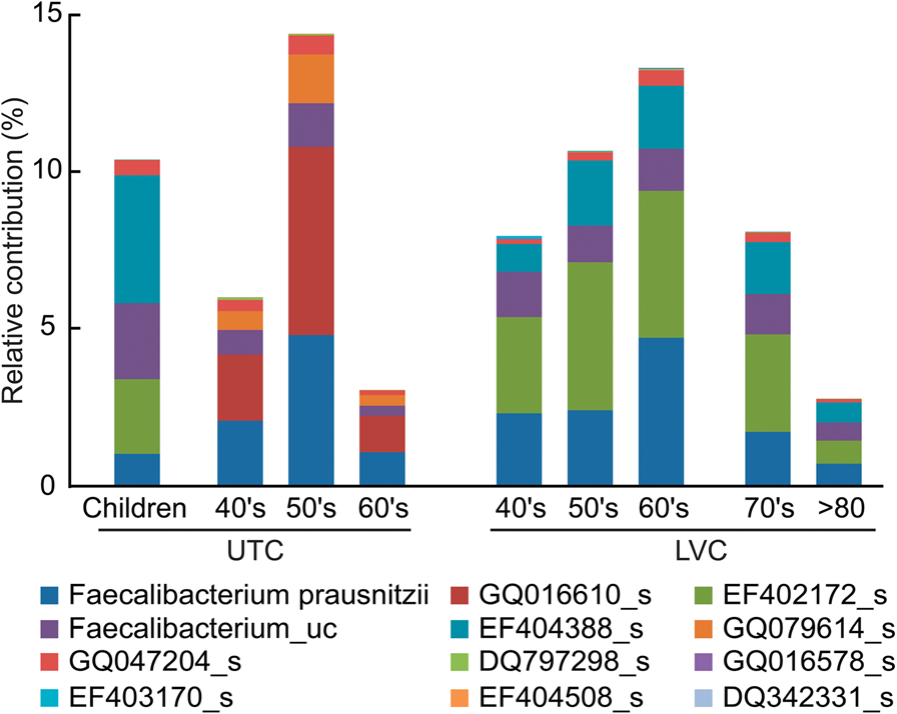
**Differences in** ***Faecalibacterium*** **spp. between UTC and LVC samples.** The relative contribution of *Faecalibacterium* spp. identified from 16 s rRNA amplicon sequencing data is shown. LVC, longevity village communities; UTC, urbanized town communities.

**Figure 3:**
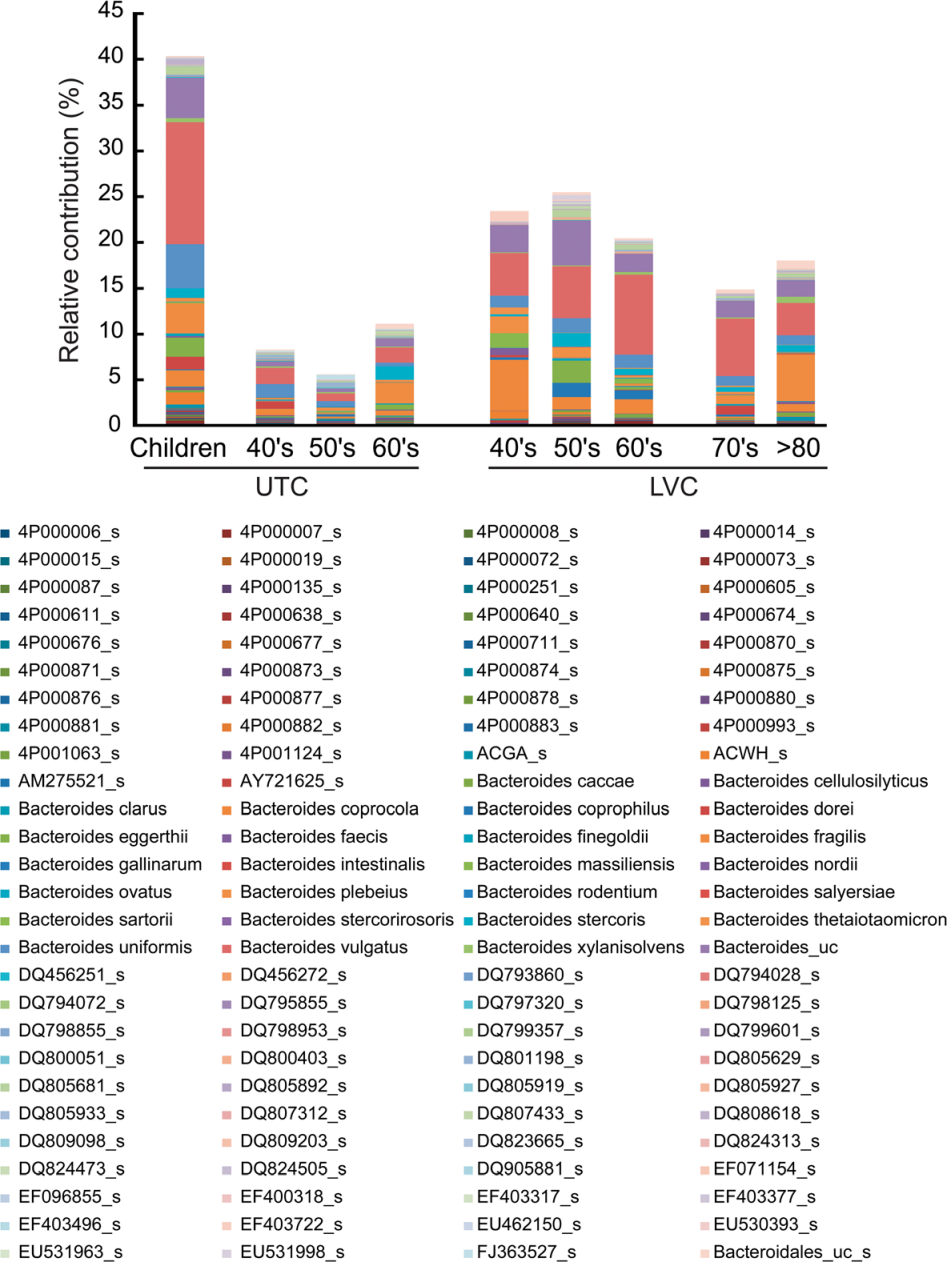
**Differences in** ***Bateroides*** **spp. between UTC and LVC samples.** The relative contribution of *Bateroides* spp. identified from 16 s rRNA amplicon sequencing data is shown. LVC, longevity village communities; UTC, urbanized town communities.

We analyzed the differences in the composition of LVC and UTC intestinal microbiota at the species level (Table 3). Most of the species detected in LVC samples were detected in UTC samples. The relative abundance of *Prevotella copri, Bacteroides vulgatus,* EU461603_s, and *Bacteroides_uc,* in LVC was significantly higher than that in UTC (40–69 y, p < 0.05), but those of *Ruminococcaceae_uc_s, EU768617_s* (*Ruminococcaceae*)*, Clostridiales_uc_s,* and *Eubacterium rectale* in LVC were lower than those in UTC. Interestingly, among *Faecalibacterium* spp., EF402172_s and EF404388_s were detected as main components in LVC (>3.9% of total sequence). These species were detected in the gut microbiota of children and LVC but not in UTC adult samples. In contrast, GQ016610 (>1.15% of total sequence) was detected as a main component in UTC adults samples, but it was not in LVC samples or children (Figure 2). Among *Bacteroides* spp., FJ363527_s, 4P000677_s, and 4P000015_s were detected in UTC samples only, but ACWH_s, EF403317_s, and EF403722_s were detected in children and LVC samples (Figure 3). Additionally, DQ905718_s (Prevotella spp.) and AY305316_s (Clostridium spp.) were detected only in children and LVC samples while 4P001066_s (Prevotella spp), 4P000016_s (Eubacterium spp.), DQ905574_s (Clostridium spp.), GQ073186_s (Streptococcus spp.), and AM277319_s (Ruminococcus spp.) were found only in UTC (data not shown).

**Table 3:** **Difference in the composition (percent of total sequences) of fecal bacterial species isolated from UTC and LVC samples**

**Species**	**Composition** ^*^ **(%)**			
	**UTC**	**LVC**		
	**Children**	**40-69 y**	**40-69 y**	**>70**
*Prevotella copri*	7.86 ± 18.92^ac^	2.81 ± 5.59^a^	8.10 ± 12.38^bc^	8.96 ± 11.43^bc^
*Dialister succinatiphilus*	0.58 ± 2.71^a^	7.19 ± 14.98^bc^	4.84 ± 8.72^bc^	2.91 ± 6.90^ac^
*Bacteroides vulgatus*	13.73 ± 15.11^a^	1.04 ± 1.92^b^	8.20 ± 10.96^ad^	5.18 ± 10.16^cd^
*Faecalibacterium prausnitzii*	1.06 ± 1.34^a^	2.81 ± 4.66^b^	2.78 ± 3.74^bc^	1.22 ± 2.15^c^
*Ruminococcaceae_uc_s*	1.53 ± 1.82^a^	3.17 ± 3.07^b^	1.94 ± 1.70^a^	2.31 ± 2.00^ab^
*EU768617_s*	0.68 ± 1.10^a^	3.15 ± 3.44^b^	0.37 ± 0.44^a^	0.43 ± 1.03^a^
*GQ016610_s*	nd^a^	3.39 ± 4.40^b^	nd^a^	nd^a^
*EU461603_s*	1.26 ± 3.64^ac^	1.14 ± 1.96^ab^	2.94 ± 4.31^c^	3.02 ± 4.80^c^
*Bacteroides_uc*	4.14 ± 3.24^a^	0.57 ± 0.83^b^	3.03 ± 3.63^ad^	1.80 ± 1.71^cd^
*Clostridiales_uc_s*	0.57 ± 0.61^a^	1.42 ± 1.12^b^	0.90 ± 0.78^c^	1.46 ± 1.46^b^
*Eubacterium rectale*	0.27 ± 0.57^a^	1.82 ± 2.38^b^	0.65 ± 1.47^a^	0.52 ± 1.11^a^
*Bacteroides fragilis*	2.96 ± 6.14^a^	0.37 ± 1.33^a^	0.20 ± 0.71^a^	2.72 ± 12.77^a^
*Prevotella_uc*	0.43 ± 0.94^a^	0.66 ± 2.31^ac^	1.42 ± 2.41^bc^	3.20 ± 5.27^b^
*EF402172_s*	2.48 ± 4.05^a^	nd^b^	4.00 ± 5.11^ad^	1.69 ± 2.98^cd^
*DQ905034_s*	1.20 ± 3.49^a^	0.98 ± 1.75^a^	0.81 ± 1.34^a^	0.84 ± 1.14^a^
*Bacteroides coprocola*	1.82 ± 5.21^a^	0.33 ± 0.81^a^	1.91 ± 5.35^a^	0.44 ± 2.12^a^
*EF401882_s*	0.80 ± 2.21^a^	0.95 ± 2.16^a^	0.44 ± 0.93^a^	1.04 ± 3.41^a^
*Escherichia coli group*	0.21 ± 0.54^a^	0.78 ± 3.16^ab^	1.77 ± 4.25^ac^	3.27 ± 9.16^bc^
*Bacteroides uniformis*	5.00 ± 4.90^a^	0.87 ± 2.61^ab^	1.59 ± 3.18^ac^	1.07 ± 2.04^bc^
*Faecalibacterium_uc*	2.54 ± 4.28^a^	0.91 ± 1.16^bc^	1.23 ± 1.23^ac^	0.96 ± 1.66^bc^
*Roseburia inulinivorans*	0.70 ± 1.75^ac^	0.56 ± 0.65^a^	1.60 ± 2.20^bc^	1.61 ± 2.69^bc^
*Megamonas rupellensis*	0.28 ± 0.73^ab^	1.43 ± 4.34^b^	0.10 ± 0.32^ab^	0.006 ± 0.03^a^
*EF404805_s*	0.41 ± 0.74^a^	1.04 ± 1.63^bc^	0.80 ± 0.87^ac^	0.99 ± 1.04^bc^
*EF405492_s*	0.09 ± 0.41^a^	1.11 ± 4.90^a^	nd^a^	0.04 ± 0.25^a^
*EU475206_s*	0.52 ± 2.43^a^	0.75 ± 2.37^a^	0.75 ± 1.95^a^	1.14 ± 1.94^a^
*Lachnospiraceae_uc_s*	0.37 ± 0.40^a^	1.10 ± 1.20^b^	0.81 ± 0.69^b^	1.02 ± 0.93^b^
*EF403870_s*	0.55 ± 1.25^a^	0.54 ± 1.05^bc^	0.77 ± 0.72^ac^	1.41 ± 2.19^bc^
*AM275436_s*	0.02 ± 0.11^a^	1.36 ± 3.26^b^	0.20 ± 0.71^a^	0.22 ± 0.74^a^
*EF404388_s*	4.26 ± 8.22^a^	nd^b^	1.64 ± 1.88^a^	1.23 ± 1.97^a^
*Dialister invisus*	1.90 ± 5.06^a^	0.91 ± 3.97^a^	nd^a^	0.23 ± 0.76^a^

^*^, Mean ± SD, data with different superscript letters are significantly different (P<0.05) according to ANOVA one-way statistical analysis.

LVC, longevity village communities; UTC, urbanized town communities; nd, not detected.

**Figure 4:**
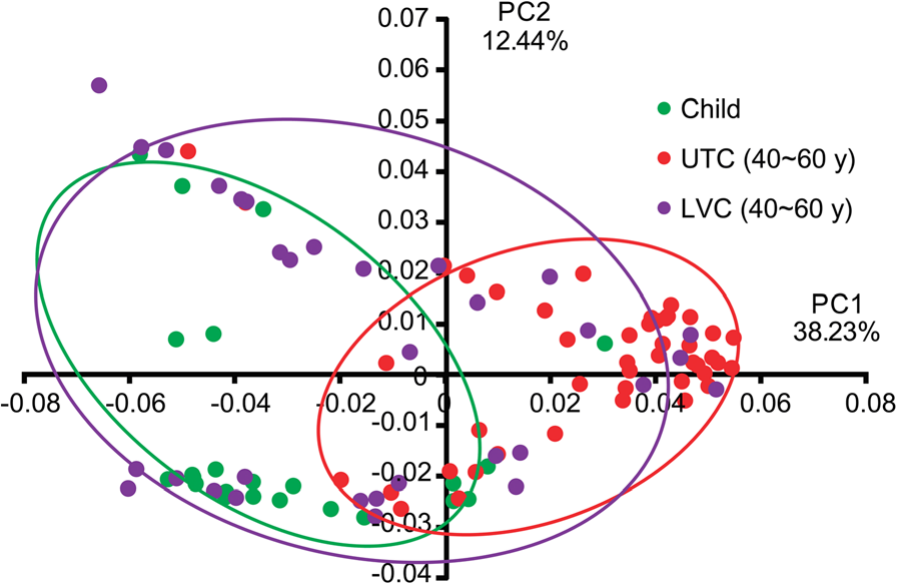
**Principal coordinate analysis plot.** The plot shows the clustering pattern between UTC and LVC based on a Principal Coordinates Analysis (PCoA). The distance matrix was calculated using weighted pairwise Fast UniFrac. LVC, longevity village communities; UTC, urbanized town communities. Green circle, child; red circle, UTC 40–69 y; purple circle, LVC 40–69 y.

Sequences of the gut microbiota 16S rRNA genes were aligned by length and position. Pairwise distances were computed for the results from LVC, UTC, and children, and principal coordinate analysis was performed to cluster the communities along axes of maximal variance (Figure 4). The gut microbial community of each group member was clustered, and the maximum variations were 38.226% (PCo1) and 12.436% (PCo2). No differences were observed between LVC samples and children. However, bacterial communities between LVC and UTC and between children and UTC showed distinguished patterns, as demonstrated by the Firmicutes to Bacteroidetes ratios. Gut microbiota compositions were segregated on PCo2 by principal coordinate analysis.

**Figure 5:**
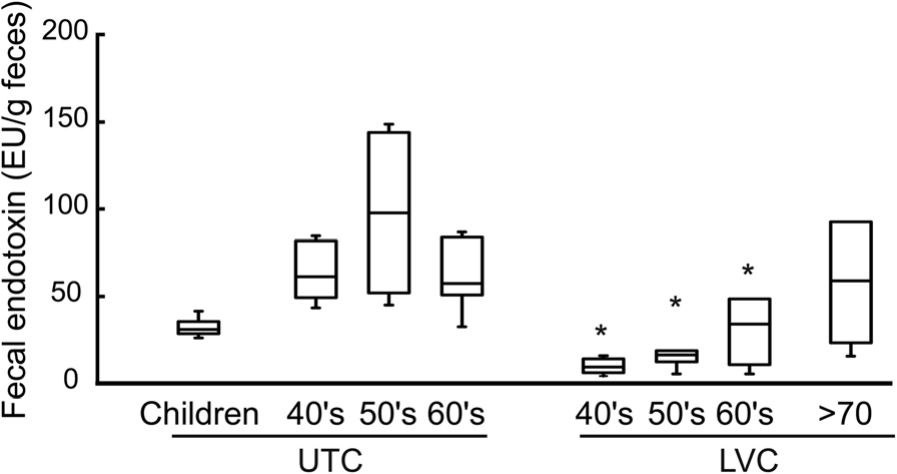
**The fecal endotoxin levels.** LAL assay was used to measure the fecal endotoxin concentration (EU/g feces). All values are indicated as the mean ± SD. *, *p* < 0.05 compared with UTC adults. LVC, longevity village communities; UTC, urbanized town communities.

Next, to understand the relationship between *Faecalibacterium* spp., known to have anti-inflammatory effect [20], and the levels of gut microbiota LPS which could induce inflammatory response, we determined fecal LPS levels in LVC and UTC adults as well as children samples. As shown in Figure 5, the fecal LPS content was significantly higher in UTC adult samples than LVC adult samples. Furthermore, the fecal LPS level was lower in children samples than in LVC samples. The correlation coefficient between the sum of EF402172_s and EF404388_s and LPS was −0.87 suggesting a strong negative linear relationship.

## Discussion

All surfaces of the human body, including the skin, mucosal surfaces, and the genital and gastrointestinal tracts are occupied by habitat-specific microorganisms. During adult life, the composition of gut microbiota is relatively stable. The colon contains 10^11^ to 10^12^ microbial cells per gram. The community composition of gut microbiota could be influenced by host genetics, health, diet, ageing, and probiotics [5,6]. In culture studies of gut microbiota related to ageing, Mitsuoka found that *bifidobacteria* declined in elderly people compared with younger adults, whereas *C. perfringens*, *Lactobacilli*, and *Enterococci* increased [21]. Hopkins *et al*. found that *Bifidobacterium* and *Lactobacillus* were lower in elderly people than in younger adults, whereas *Bacteroides* and *Eubacterium* were the same [22]. Health-promoting bacteria such as *bifidobacteria* have been thought to decrease with advancing age, but the most recent studies based on high-throughput sequencing techniques contradict these previous reports [23,24]. Nevertheless, levels of facultative anaerobes, including *streptococci, staphylococci, enterococci,* and *enterobacteria* were higher in the elderly than in younger adults.

Recently, it has been reported that Clostridium cluster XIVa, one of the Firmicutes phyla, was decreased in Japanese, Finnish, and Italian elderly and centenarians, whereas it was increased in German older adults [24-27]. *Faecalibacterium prausnitzii*, belonging to the Clostridium cluster IV, was markedly decreased in Italian elderly and centenarians, but this result was not confirmed in other European populations [26]. In addition, *Bacteroidetes* was increased in German, Austrian, Finnish, and Irish elderly, but not in Italian elderly and centenarians.

In this study, most of the identified microorganisms belonged to the Bacteroidetes and Firmicutes phyla. Proteobacteria, Fusobacteria, and Actinobacteria phyla were less than 10% of the total community, similar to previous reports [28]. Therefore, at the phylum level, the community composition of Korean gut microbiota was similar to compositions previously reported for other human populations [29]. In the present study, we found that the Firmicutes to Bacteroidetes ratio in UTC adults was higher than that in LVC adults and children. Interestingly, we found no difference in the Firmicutes to Bacteroidetes ratio between children and LVC. These changes are consistent with the results from the principal coordinate analysis.

In the present study, anthropometric characteristics and eating as well as smoking habits were not different in both adult samples. However, according to the study by Yon et al. [30] which investigated food consumption and dietary pattern in middle aged and older adults (45–93 years in male, 45–105 years in female) living in Gugoksundam (Gurye, Goksung, Sunchang and Damyang counties) longevity area where we selected the LVC adults for the present study, the average total daily food intake was significantly decreased with aging and animal food intake ratio to total food intake was ranged 10–14 % which is significantly lower than the Korean average animal food intake reported in The Korea National Health and Nutrition Examination Survey in 2007. Furthermore, Kim et al. [31] reported that the intake of vegetables in the rural elderly was significantly higher than in the urban elderly when they performed the survey to evaluate dietary behavior, food intake, and satisfaction with food-related life between the elderly living in urban and rural areas of Korea. In addition, the rural elderly had better balanced diet and were more satisfied with food related life than those of the rural elderly. These results suggest that animal food and vegetable intakes may alter the gut microbiota composition.

We also found that the compositions of *Faecalibacterium* spp. and *Bacteroides* spp. in UTC and LVC samples were different. Although the levels of *Faecalibacterium prausnitzii* in UTC and LVC were not significantly different, EF402172_s and EF404388_s, belonging to *Faecalibacterium* spp. were the predominant intestinal microbiota (>3.9%) identified in LVC samples but not UTC samples. Some *Bacteroides* spp., ACWH_s, EF403317_s, and EF403722_s, were detected only in LVC samples, but they constituted less than 0.5% of the gut microbiota. Some intestinal bacteria, 4P001066_s (*Prevotella* spp.), 4P000016_s (*Eubacterium* spp.), DQ905574_s (*Clostridium* spp.), GQ073186_s (*Streptococcus* spp.), and AM277319_s (*Ruminococcus* spp.) were detected only in UTC samples. In these conditions, the fecal LPS content was significantly higher in UTC adults than LVC adults. These results suggest that the increased levels of gut microbiota LPS by increased animal food intake, decreased vegetable intake, and increased and/or only detected intestinal bacteria in UTC adults may induce unresolved chronic inflammation which is described as an underlying mechanism of aging and age-related diseases reviewed in [32]. In addition, the anti-inflammatory *Faecalibacterium* spp. EF402172_s and EF404388_s might play an important role in preserving individuals’ health in LVC by inhibiting the production of LPS. In this study, the comparative analysis of gut microbiota in children and elderly people of urbanized towns and longevity villages suggest that maintaining gut microbiota of healthy children with dietary interventions could be beneficial to promote healthier ageing.

## Conclusions

Overall, the gut microbiota composition and LPS levels of the elderly people of UTC were significantly different from that of the elderly community of LVC, which is quite similar to that observed in UTC children. Among *Bacteroides* spp., ACWH_s, EF403317_s, and EF403722_s were detected in LVC and children samples only. EF402172_s and EF404388_s, members of *Faecalibacterium* spp., which are known to have anti-inflammatory properties, were detected in LVC and children samples only (>3.9% of total sequence). In addition, the fecal lipopolysaccharides (LPS) content was significantly higher in UTC than in LVC. Our findings suggest that the alteration in the composition of gut microbiota and decrease in LPS levels provably driven by diet may play an important role in preserving residents’ health in LVC.

## Additional files

Additional file 1: Table S1. Number of sequences analyzed, observed diversity richness (OTUs), estimated OTU richness (ACE and Chao1), and coverage. Additional file 2: Figure S1. Rarefaction curves. Rarefaction analysis of V1-V3 pyrosequencing tags of the 16S rRNA gene in fecal microbiota from UTC and LVC. **(A)** UTC children, **(B)** 40′s in UTC and LVC, **(C)** 50′s in UTC and LVC, **(D)** 60′s in UTC and LVC, **(E)** over 70 in LVC. LVC, longevity village communities; UTC, urbanized town communities.
