# Supplementary material for: Comparative analysis of gut microbiota in elderly people of urbanized towns and longevity villages
Source: BMC Microbiol. 2015 Feb 26;15:49. doi: 10.1186/s12866-015-0386-8 (PMC4345030; doi:10.1186/s12866-015-0386-8)
Supplement: Additional file 1: Table S1. — Number of sequences analyzed, observed diversity richness (OTUs), estimated OTU richness (ACE and Chao1), and coverage. [file 12866_2015_386_MOESM1_ESM.doc]

**Table S1. Number of sequences analyzed, observed diversity richness (OTUs), estimated OTU richness (ACE and Chao1), and coverage.**

|  |  |  | Phylotype | | |  |
| --- | --- | --- | --- | --- | --- | --- |
| Group | age | Total reads | OTUs | ACE | Chao1 | Goods Coverage |
| UTC | Children | 5218±3730 | 426±358 | 935±795 | 741±627 | 0.96±0.03 |
|  | 40’s | 8201±3464 | 742±391 | 1363±824 | 1169±662 | 0.95±0.03 |
|  | 50’s | 8394±4006 | 877±359 | 1565±677 | 1363±579 | 0.95±0.02 |
|  | 60’s | 8837±3772 | 800±545 | 1553±1333 | 1256±963 | 0.96±0.02 |
| LVC | 40’s | 3605±2625 | 279±112* | 516±225* | 435±148* | 0.96±0.01 |
|  | 50’s | 6843±4065 | 651±445 | 1335±1071 | 1090±769 | 0.96±0.03 |
|  | 60’s | 7562±3887 | 503±242 | 846±418 | 759±351 | 0.97±0.03 |
|  | >70 | 5647±3760 | 621±426 | 1557±1750 | 1225±1013 | 0.94±0.05 |

The cutoff value of the phylotype is equal to or greater than 97% similarity.

All values are the mean±SD. OTUs, operational taxonomic units; LVC, longevity village communities; UTC, urbanized town communities

*, *p*>0.05 compared to UTC, 40’s
