# Supplementary figures and images for: Comparative analysis of gut microbiota in elderly people of urbanized towns and longevity villages
Source: BMC Microbiol. 2015 Feb 26;15:49. doi: 10.1186/s12866-015-0386-8 (PMC4345030; doi:10.1186/s12866-015-0386-8)

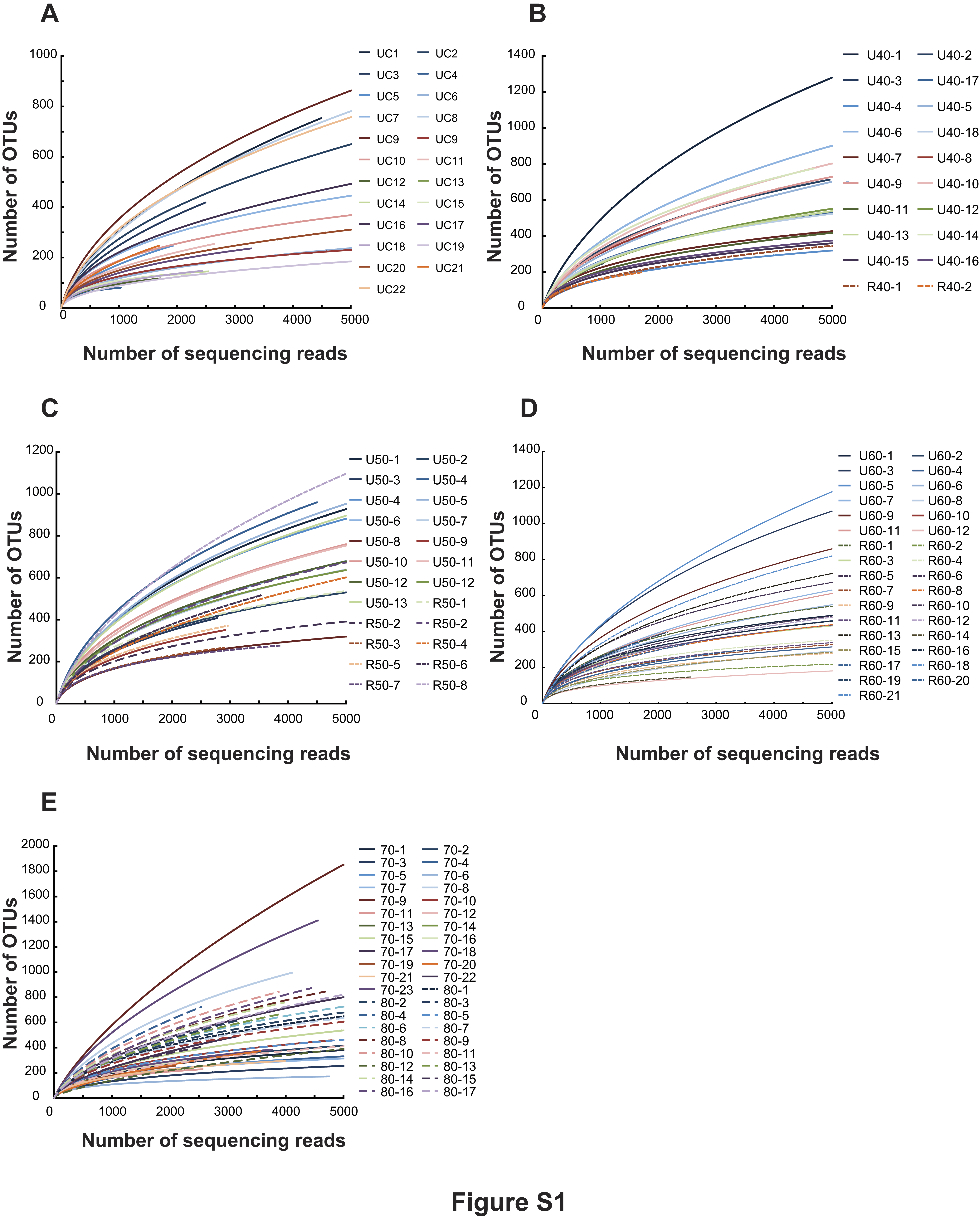

Supplement: Additional file 2: Figure S1. — Rarefaction curves. Rarefaction analysis of V1-V3 pyrosequencing tags of the 16S rRNA gene in fecal microbiota from UTC and LVC. (A) UTC children, (B) 40′s in UTC and LVC, (C) 50′s in UTC and LVC, (D) 60′s in UTC and LVC, (E) over 70 in LVC. LVC, longevity village communities; UTC, urbanized town communities. [file 12866_2015_386_MOESM2_ESM.tiff]
